# Professional Oversight of Emergency-Use Interventions and Monitoring Systems: Ethical Guidance From the Singapore Experience of COVID-19

**DOI:** 10.1007/s11673-022-10171-1

**Published:** 2022-04-14

**Authors:** Tamra Lysaght, Gerald Owen Schaefer, Teck Chuan Voo, Hwee Lin Wee, Roy Joseph

**Affiliations:** 1 Centre for Biomedical Ethics, Yong Yoo Lin School of Medicine, Clinical Research Centre MD 11 #02-03, National University of Singapore, 10 Medical Drive, Singapore, Singapore; 2 Saw Swee Hock School of Public Health, National University of Singapore, Singapore, Singapore; 3 Department of Pharmacy, Faculty of Science, National University of Singapore, Singapore, Singapore; 4 Department of Paediatrics, National University Hospital, Singapore, Singapore

**Keywords:** COVID-19, Emerging communicable diseases, Ethical framework, Professional ethics

## Abstract

High degrees of uncertainty and a lack of effective therapeutic treatments have characterized the COVID-19 pandemic and the provision of drug products outside research settings has been controversial. International guidelines for providing patients with experimental interventions to treat infectious diseases outside of clinical trials exist but it is unclear if or how they should apply in settings where clinical trials and research are strongly regulated. We propose the Professional Oversight of Emergency-Use Interventions and Monitoring System (POEIMS) as an alternative pathway based on guidance developed for the ethical provision of experimental interventions to treat COVID-19 in Singapore. We support our proposal with justifications that establish moral duties for physicians to record outcomes data and for institutions to establish monitoring systems for reporting information on safety and effectiveness to the relevant authorities. Institutions also have a duty to support generation of evidence for what constitutes good clinical practice and so should ensure the unproven intervention is made the subject of research studies that can contribute to generalizable knowledge as soon as practical and that physicians remain committed to supporting learning health systems. We outline key differences between POEIMS and other pathways for the provision of experimental interventions in public health emergencies.

## Introduction

High degrees of uncertainty and a lack of specific effective treatments have characterized the COVID-19 pandemic since its emergence in early 2020. A public health emergency of international concern (PHEIC), the pandemic triggered the need to initiate clinical trials on potential medical interventions expeditiously. Well-designed clinical trials remain the primary way to develop robust generalizable evidence for potential treatment modalities even during a PHEIC. In addition, like all formal research, they offer patients receiving unproven interventions the protection of research ethics requirements such as benefit-risk assessment by an ethics review committee. Reduction of suffering is of course a general ethical obligation in biomedicine, including during a PHEIC (Nuffield Council on Bioethics 2020). In a context where many patients are dying or suffering from a novel disease such as COVID-19, physicians may seek to fulfil this obligation by providing unproven interventions, which may include off-label or experimental therapies, to their patients outside a controlled trial setting. Such provision may be ethically appropriate under certain circumstances and when it observes certain ethical norms (e.g. favourable benefit-risk ratio) and processes (e.g. pharmacovigilance). For example, off-label use of medications (i.e. use of approved drugs for a condition or administered in a way or in a dose that is not in the approved label) may be given to patients on *a case-by-case* basis if it is in their best interest, when opportunities for clinical research are not available and if the prescription adheres to national laws and regulations (Stolbach et al. 2020).

Widespread provision of unproven interventions outside clinical trials through off-label prescription or other pathways such as expanded access or compassionate use, which has happened in many countries particularly in the earlier stages of the COVID-19 pandemic, is ethically controversial. Expanded access is a protocol or regulatory mechanism that permits the use of an investigational medical product outside of a clinical trial by patients with a serious or life-threatening condition who have no viable treatment options available to them, and who are ineligible or unable to participate in the clinical trial in progress (Jarow et al. 2017). Uncontrolled access to unproven interventions through concurrent and disparate clinical and regulatory routes outside clinical trial settings may derail or prolong clinical trials by diverting resources away. It may also confuse clinicians and the public on the benefit-risk profiles of the interventions which, in turn, may increase the pressure for non-trial access and undermine trial initiation or recruitment or potentially undermine trial consent and trust in research (National Academies of Sciences Engineering and Medicine 2017).

### International Ethical Frameworks

**Figure 1:**
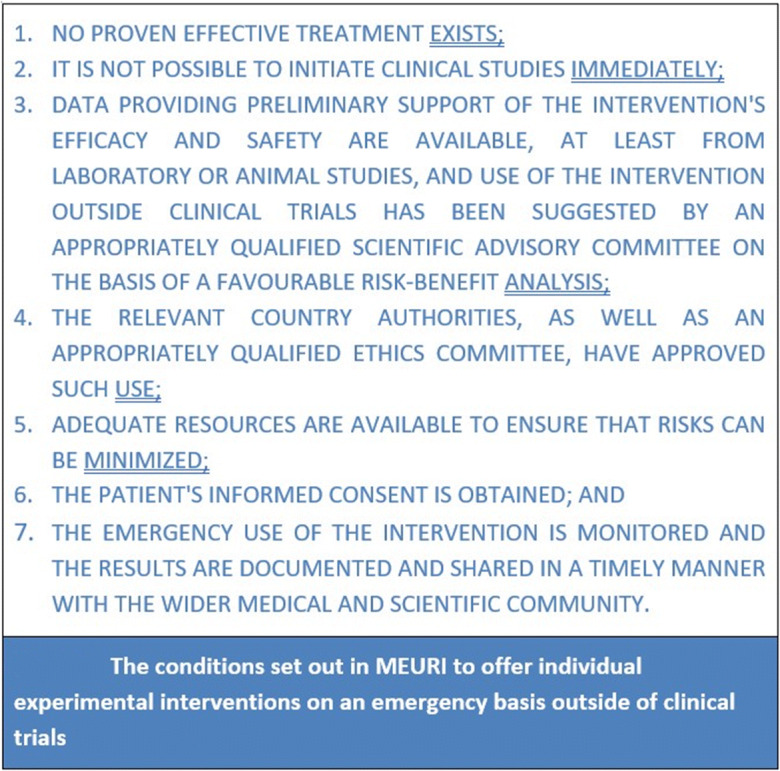
The conditions set out in MEURI to offer individual experimental interventions on an emergency basis outside of clinical. Created by the authors

As promulgated by the WHO (2016), academic commentators, and various national health authorities, unproven interventions should be provided to COVID-19 patients either through clinical trials or through the MEURI (Monitored Emergency Use of Unregistered and Investigational Interventions) framework (Cortegiani et al. 2020; Pan American Health Organization 2020; World Health Organization 2020; Thirion and Lau 2020; Zuckerman et al. 2021). Developed by the expert panel convened by the WHO in response to the 2014-16 Ebola epidemic in West Africa, MEURI was applied during the West African Ebola epidemic, the 2018 (ongoing) Ebola epidemic in the Democratic Republic of the Congo, as well as in other emergency contexts (WHO 2016). MEURI may be described as an ethical framework with a set of conditions (see Figure 1) for developing a protocol to monitor the use of unproven medical interventions outside of research for therapeutic or preventive purpose during a public health emergency (Mastroleo, Smith, and The WHO MEURI Working Group 2020). With focus on the Americas region, the Pan American Health Organization (PAHO 2020) issued a document “Emergency use of unproven interventions outside of research: Ethics guidance for the COVID-19 pandemic” to advance the use of MEURI and clarify its ethical criteria by categorizing them into features of justification; ethical and regulatory oversight; consent process; and contribution to the generation of evidence.

Notably, PAHO’s (2020) document highlighted some challenges with MEURI’s application during COVID-19 in the Americas and elsewhere, which includes lack of familiarity with the framework leading to non-adherence to one or many of its criteria. According to PAHO (2020), MEURI may also be wrongly conflated with observational research, which may be due to common features between MEURI and research such as the requirement of ethics review and approval, albeit not necessarily by an institutional review board or research ethics committee (REC), and contribution to the production of evidence or knowledge, viz. monitoring, documenting, and sharing its results in a timely manner with the wider medical and scientific community. As PAHO (2020) notes, MEURI is not research and is to be applied only under exceptional circumstances i.e. access to unproven interventions outside clinical trials during an emergency situation should be provided only when clinical studies are unavailable or infeasible to initiate because of reasons such as an overwhelmed health system, lack of research capability or resources, insufficient patient numbers, and so forth. Nevertheless, on the basis that MEURI “should be guided by the same ethical principles that govern the use of unproven interventions in clinical trials,” PAHO (2020, 3) states that MEURI protocols *must be* reviewed by a REC.

PAHO’s (2020, 4) further recommendation that “MEURI must contribute to the generation of knowledge” could lead MEURI to be defined as research in some jurisdictions, triggering various regulatory requirements. Given this implication, as well as other considerations, such as lack of available mechanism for the registration of MEURI protocols and systematic collection of its data, it may be simpler to do formal research. Indeed, countries or health systems with strong infrastructural separation between research and clinical practices may prefer to simply conduct observational research or establish a registry rather than deploy MEURI to generate preliminary data on the safety and efficacy of unproven interventions. Alternatively, they may also opt for other pathways to provide cohorts of patients with unproven interventions outside of clinical trials, which lack the public good feature of contribution to evidence generation during a PHEIC.

This paper considers the application of frameworks for the provision of unproven interventions outside clinical trials during a PHEIC in countries with well-developed regulatory infrastructure for human subject research. Drawing on our experience developing guidance for the provision of experimental or non-standard interventions to treat COVID-19 in Singapore,^1^ we propose the Professional Oversight of Emergency-Use Interventions and Monitoring System (POEIMS) as an ethical alternative for these settings. The city-island state of Singapore has a comprehensive legal framework that regulates all biomedical research with human subjects with requirements for ethics review of activities that are intended to contribute to generalizable knowledge. While contributing to the knowledge base with well-designed clinical trials investigating on safety and efficacy is ideal and should remain the priority, (London and Kimmelman 2020; London 2021) denying access to an experimental intervention may conflict with the physicians’ duty of care to act in their patients’ best interests.

We contend that such interventions may be provided to patients outside formal research settings in very limited circumstances where systems exist for monitoring and evaluating the use of unproven interventions during a public health emergency involving a novel infectious disease. To support this argument, we establish moral duties for physicians to record meaningful outcomes data and for institutions to establish monitoring systems for reporting information on safety and effectiveness to the relevant national and international authorities. Institutions should also commit to supporting learning health systems, ensuring that the provision of unproven interventions does not impede the conduct of clinical research and that the intervention is made the subject of clinical studies that can contribute to generalizable knowledge as soon as practical. We conclude with an overview of key differences between our POEIMS proposal and that of observational research, MEURI, and expanded access programmes (summarized in Table 1).

**Table 1:** Comparison between frameworks for expanded access (based on FDA conditions), MEURI and POIEMS

	Expanded Access	MEURI	POEIMS
Patient eligibility	Immediately life-threatening or serious disease	Unspecified (though MEURI is to be applied in an emergency context marked by high mortality)	May apply to patients with immediately life-threatening, serious, and moderate disease
	No treatment or research options (including eligibility for ongoing clinical trials)	No proven effective treatment option, and no research option (as there is no existing clinical trial in the given setting)	No proven effective treatment option, and no research option (as there is no existing clinical studies in the given setting)
Required support	Treating physician IRB Relevant regulatory authority for drugs and other health products Manufacturer or sponsor	Appropriately qualified scientific advisory committee Appropriately qualified ethics committee (RECs or IRBs, as recommended by PAHO) Relevant regulatory authority for drugs and other health products	Professional consensus Appropriately qualified and constituted CECs or other hospital ethics committees Relevant regulatory authority for drugs and other health products
Goals of monitoring and documentation and reporting of safety and efficacy outcomes	For protection of patient safety, widening of expanded access, and accelerating drug/product approval, by manufacturers and the scientific and regulatory authorities	For protection of patient safety and contribution to evidence generation by the wider medical and scientific community	For protection of the safety of the patient receiving the intervention and future treatment applications in the emergency context, and ancillary use for the consideration of the initiation of clinical studies by the relevant hospital, scientific and regulatory authorities.

## The Singapore Experience with COVID-19

The first imported case of COVID-19 in Singapore was recorded on January 23, 2020 and locally transmitted cases on February 4, 2020. The Disease Outbreak Response System Condition (DORSCON) was raised to Orange (the third highest level of four) shortly afterwards. However, it was not until a surge in locally transmitted cases during April that the Singapore government imposed a national-wide “circuit breaker” lockdown (Lee 2020). At that time, Singapore had 1,098 active cases with twenty-nine patients in intensive care (Ministry of Health 2020b). With large outbreaks in the foreign worker dormitories, the active case load peaked in May at just over 20,000, although the number of patients admitted to ICU remained relatively low at <30 (Ministry of Health 2020a). While cases remained very low throughout 2020 until mid-2021, outbreaks of the Delta and Omicron variants have since increased infections despite high rates of vaccination and rising hospitalizations have placed greater pressure on the health system. However, as of April 2022, the death rate remained relatively low at <0.2 per cent (Ministry of Health 2022).

Reasons for the low death rate in Singapore even before widespread vaccination are likely due to multiple factors, including pandemic preparedness, prompt and effective public health response to the dormitory outbreaks amongst a relatively young group of immigrant workers, the early detection of those with moderate to severe disease, through objective and systematic monitoring of those with mild illness in urgently deployed community care facilities, and the management of those with moderate and severe disease in non-overwhelmed tertiary care facilities (Tan et al. 2020). In the early phase of the epidemic in Singapore, the National Centre for Infectious Diseases (NCID) convened a Therapeutic Workgroup to issue guidelines for the diagnosis and treatment of patients according to disease severity (Vasoo et al. 2020). These guidelines were developed at a time of great uncertainty around the natural progression of COVID-19 and with no known effective therapeutic agent against the virus itself.

At the same time, guidance was needed on the use of non-standard and experimental interventions, such as those being investigated overseas and in locally conducted trials (e.g. with Gilead’s remdevisir). While the default position rightly restricted the use of these interventions to clinical trials, it was recognized that there would be situations where some patients would be unable to gain access to them—either because trials were not being conducted locally or, if they were, an individual patient may not meet the eligibility criteria or simply may not wish to participate, for whatever reason. Additionally, some medical practitioners wanted the freedom to prescribe certain market approved medications “off-label” (e.g. hydroxychloroquine) and immunotherapy products (e.g. interleukin inhibitors and convalescent plasma) outside clinical trials. There were also reports of patients (or their families) requesting the importation of highly experimental stem cell-based products as an innovative therapy.

### Existing Ethical Guidelines

Applying MEURI described above would be legally and ethically problematic within the Singapore regulatory context. Notably, the requirement to contribute to knowledge production through the monitoring, documenting, and sharing of results “in a timely manner with the wider medical and scientific community” (as stipulated in point seven of MEURI—see Table 1), may be viewed as an activity that local legislation defines as research. The Singapore *Human Biomedical Research Act (HBRA) 2017* defines research as “any systematic investigation with the intention of developing or contributing to generalisable knowledge” (section 2, 8). Whether monitoring, documenting, and sharing results with the scientific and medical community constitutes the development or contribution to *generalizable knowledge* is unclear, but if such activities fall within the scope of the law, they would require ethics review from an institutional review board (IRB). Compliance with this aspect of MEURI in Singapore, particularly as per PAHO (2020) recommendations for REC review, may thus restrict the provision of an unproven intervention for COVID-19 patients to the context of an IRB-approved research protocol.

Such protocols would not be limited to clinical trial design. They could be an observational study or clinical registry to record and disseminate patient outcomes data with the research community, but they would nonetheless require the submission and approval of a written research protocol with risk-benefit assessments, defined eligibility criteria, data management plans, reporting and insurance provisions, informed consent procedures, and so on. This requirement may delay the availability of an experimental intervention by weeks to months depending on the expeditiousness of the IRB assigned to review the protocol. On the one hand, this delay may be viewed as inappropriately withholding potentially effective treatments to patients in an emergency. On the other hand, it may reduce the risk of patients being inappropriately treated with interventions that prove to be unsafe. From anecdotal reports, and the authors’ experience, IRBs in Singapore have been expediting reviews and producing relatively fast approvals for research protocols studying COVID-19 throughout the pandemic.

Even so, the 2016 Ethical Code and Ethical Guidelines (ECEG) of the Singapore Medical Council allow physicians to not only prescribe medications off-label but to also provide unproven interventions outside the context of formal research under certain circumstances. According to the Section B5 (9) of the ECEG, physicians may prescribe medications off-label providing “it is in the patients’ best interests, there is rational basis, patients have justifiable medical indications, [and] you have assessed the risks and benefits of such use” (Singapore Medical Council 2016a, 27). In addition to proper documentation of consent, the SMC Handbook on Medical Ethics (an educational resource accompanying the ECEG) advises that patients receiving off-label treatments “should be appropriately monitored for effectiveness and side effects” (Singapore Medical Council 2016b, sB5.8, 55). In Singapore, healthcare institutions have policies/processes for monitoring and reporting adverse side effects of medications that are prescribed off-label.

According to these guidelines, physicians could thus prescribe at least some off-label or untested medications for COVID-19 patients providing they and their institutions can meet these conditions. It is only when the variances from standard use are “so significant that they render the techniques novel and unclear in their risk profiles” (Singapore Medical Council 2016a, SB6(2), 28) or “significantly increase the risks (or degree of ignorance of risks),” that the intervention should be limited to the context of “formal and approved clinical trials” that are “subject to the ethics of research” (Singapore Medical Council 2016b, sB6, 56-57). Even then, the ECEG allows for exceptional circumstances when an unproven intervention could be provided as an “innovative therapy” outside the context of an IRB-approved research protocol. According to the SMC, interventions that are “completely novel or significantly modified standard therapy with little or nothing in the way of studies or scanty evidence of efficacy, effects or side effects” may be provided when conventional therapy is unhelpful and “it is a desperate or dire situation” (Singapore Medical Council 2016b, sB6.1, 57). In those circumstances, physicians should seek professional consensus on the use of the intervention in that particular clinical situation and obtain informed consent as appropriate.

Since COVID-19 is an emergent disease that had no known effective standard of care treatments at the time of the outbreaks in Singapore, any intervention aimed at treating the disease and not just the symptoms would fall within this definition of “untested” or “unproven.” If no clinical trials or IRB-approved studies have been established in Singapore, then physicians would require guidance on when it would be ethically acceptable to provide various non-standard or experimental interventions in the treatment of moderate and severe COVID-19 disease. Hence, we set about preparing guidance that physicians could refer to in these situations where there is urgent unmet medical need and there are no formal trials available for patients to access the intervention in an IRB-approved study.

### Ethical Guidance for Treating COVID-19 Patients With POEIMS

In developing this guidance, we set aside interventions that could reasonably fall under the SMC’s (and the WHO’s) provisions for off-label prescription. That is, for medications that have well-established safety profiles to assess the relative benefits of such use in the best interest of patients with justifiable medical indications, who are able to consent, and so on. As example of such medications for COVID-19 would be dexamethasone. Even though they may lack evidence of efficacy for the treatment of COVID-19 specifically, we set these interventions aside with the assumption that the hospital or healthcare institution has established professional consensus on which patients they may be prescribed to in a written protocol and that mechanisms are in place to monitor and report adverse outcomes to the relevant authority, as would normally occur with these practices.

For interventions that would not reasonably fall under off-label provisions because their risk profile for COVID-19 patients is unclear or their use significantly increases the uncertainty of risk, the ECEG ought to apply with the following clarifications that take into account the context of practicing in a global pandemic. These interventions would include investigational drugs (e.g. Remdevisir prior to market licensure), biologics not demonstrated as safe and effective for treating COVID-19 (e.g. convalescent plasma), and other experimental products, such as mesenchymal stem cells. Patient(s) being offered these interventions should meet the criteria set out in the SMC ECEG of having no other helpful options and being “in a desperate or dire situation” (Singapore Medical Council 2016b, sB6.4). COVID-19 patients with severe disease for whom the intervention provides the opportunity for saving of life or amelioration of intolerable pain and suffering, and who are not able to enrol on an IRB-approved study for any reason, may be judged to be in a sufficiently dire situation to justify providing the intervention solely as part of the individual patient’s clinical care.

It might also be argued that this definition could extend to patients who are known to be at high risk of progressing to severe disease (based on other epidemiological indicators) but whose clinical condition is mild/moderate. On the one hand, treatment of such patients at an earlier stage in the disease might avoid more invasive interventions if the condition worsens. On the other hand, the costs of treating unknown side effects from unproven interventions may limit the overall benefits for such patients. In such cases, the uncertainties should be assessed and it must be clear that the individual patient’s best interest is served by early intervention and when the potential risks are materially lower than the likelihood of averting progression to a severe state.

Having met these criteria, there should be consensus among relevant professionals on a favourable benefit-risk ratio in the patient’s specific clinical context. The physician should be appropriately qualified to treat the disease with the novel therapeutic and provide to the institution’s hospital or Clinical Ethics Committee (CEC) on case-by-case basis a written plan outlining treatment goals, the system for monitoring and reporting outcomes, and exit criteria. The CEC is preferred over an ad hoc committee convened for the purpose reviewing such proposals because an ad hoc body would lack the continuity and training of an established CEC. It is also preferred over an institutional innovation ethics committee. Dedicated innovation committees have been introduced at some institutions, particularly for oversight of innovative surgery but are not widely utilized for reasons that are unclear (McNair and Biffl 2015); possibly because hospital ethics committees are fulfilling this role without the need for a dedicated innovation body (Castlen and Cochrane 2019). While such committees may be a viable alternative in certain institutional settings, or at least in theory, our preference for levering existing infrastructure for CECs is more pragmatic: it would not require creating an entirely new entity or membership and the process can be folded into the existing committee’s scope of work.

Consent from the patient or permission from their next-of-kin should be secured, based on relevant information on the uncertainty regarding the probability of benefits and adverse outcomes. Proper documentation should be maintained and outcomes should be reported in a timely way to the relevant national and international authorities for monitoring purposes. Although this requirement places additional demands on clinicians, who are likely to be stretched in the context of a public health emergency, it is ultimately for the benefit of patients, as elaborated on in our justifications below. If the proposed clinical goals are achieved, the intervention should be made subject of an approved IRB study as soon as practical.

To support these interventions, institutions or the relevant authorities should have in place specific requirements for notification of such plans for approval and consideration of these requests should be expedited. They should also have in place mechanisms for reporting and monitoring. Where the institution does not have any CEC or capacity to establish these mechanisms, it should either convene an expert group to consider and monitor such treatment plans or advise their physicians to refer patients to an institution that does.

Additionally, for investigational drugs or products that fall under the purview of drug regulatory authorities and have not received market licensure or an emergency use authorization, the physician or institution should apply through the appropriate channel for compassionate use or expanded access. In Singapore, institutions must apply to import and provide unauthorized products through the Special Access Route of the Health Sciences Authority (HSA). As per HSA guidance (Health Sciences Authority 2020), it must be for a specific, individual patient for life-saving treatment for which there is no alternative registered therapy available. As the HSA does not evaluate these products for quality, efficacy, and safety, the full responsibility for use of such a product, once approved, lies with the prescribing physician. The manufacturer’s protocols for patient selection, contra-indications, and administration etc. should be strictly adhered. Once systematically offered to a series of patients meeting defined criteria, they should be made the subject of an IRB-approved clinical trial at the earliest opportunity.

In the next section, we set out the justifications for POEIMS as outlined above, which essentially allows physicians to enact their duty of care to COVID-19 patients with ethical oversight while placing duties on institutions to establish reporting mechanisms to support the use of these interventions.

## The Duty of Care and Duty to Support Learning Health Systems

### Duty of Care

The primary justification for provision of unproven interventions is the best interests of the patient. Normally, shared decision-making models would aim to establish whether an intervention does indeed meet that standard through physician advice and patient reflection on the goals of care, and what is achievable given their condition and the existing available treatments. However, it would be a mistake to think such judgments exist in a vacuum. Professional advice is not based on the insights of a physician alone but is built on a larger body of peer judgment and wisdom. Evidence-based medicine involves those peer judgments, in turn, being informed by rigorous empirical studies of the treatments to establish safety and efficacy. By definition, unproven or experimental interventions lack such an evidentiary framework. Though a myriad of interventions may be “promising” when initially developed, many ultimately fail to pass standards of safety and efficacy necessary for regulatory approval (Hwang et al. 2016). There is, then, a substantial risk that a physician gets it wrong in making an unproven recommendation; that is, their individual judgment is mistaken.

The exceptional safeguards in our POEIMS proposal are needed in light of these epistemic limitations concerning unproven treatments to protect patients’ interests. Requiring a clear protocol for provision of unproven interventions helps promote rigorous, consistent applications of good practices, especially when multiple patients in similar situations may receive the same unproven treatment. In addition, review by a CEC or other institutional body provides the opportunity for broader peer input and feedback, to minimize the impact of idiosyncratic judgments of individual physicians and bring to bear a larger body of experience and expertise to inform decision-making. Our proposal may be regarded as a modification of the Declaration of Helsinki (DoH) guidance on “Unproven Interventions in Clinical Practice” (World Medical Association 2018), which is not specifically directed at public health emergencies:

In the treatment of an individual patient, where proven interventions do not exist or other known interventions have been ineffective, the physician, *after seeking expert advice*, with informed consent from the patient or a legally authorised representative, may use an unproven intervention *if in the physician’s judgement* it offers hope of saving life, re-establishing health or alleviating suffering. This intervention should subsequently be made the object of research, designed to evaluate its safety and efficacy. (37, italics ours)

Our approach differs substantially from individualistic clinical practice where therapeutic judgment of the treating physician alone—albeit informed by expert advice, as proposed by the DoH—is sufficient to justify treatment. POEIMS aims to protect patients from ineffective or harmful innovative therapies through its requirements for structured protocol and peer review.

Some have argued that restrictions like these on access to unproven treatments are unacceptably paternalistic (Brodrick 2020; Salter, Zhou, and Datta 2015). That is to say, restrictions are placed on the conditions under which patients can access unproven or experimental interventions, in the name of protecting their interests. Yet, if patients are the best-placed to assess whether a given intervention’s risk are worth its benefits, then the decision of whether to accept an unproven therapy should on this line of thinking be left up to them. This more minimalist approach would then primarily focus only on informed consent requirements for unproven therapies: be honest and open about limitations on the evidence, and the uncertain prospects of success, leaving it to patients to decide whether to accept those risks.

Minimalist approaches face several justificatory problems. Even for well-established proven treatments, epistemic limitations on patients’ understanding of treatment options and ramifications limit the extent to which consent can be said to be fully informed (Walker 2012; Boyd 2015). Consent is normally deemed necessary but not sufficient for the protection of subjects’ interests. Those epistemic limitations are even more greatly exacerbated in the case of unproven interventions, where even treating physicians are poorly placed to adequately advise on whether an intervention would likely be effective. Inability to fully evaluate an intervention’s risks and benefits limits the extent to which autonomy (as self-governance) can be realized through the consent process, and so autonomy objections to restrictions on access are less pressing in the case of unproven interventions.

In addition, whether a treatment is in a patient’s best interests is not fully determined by that patient’s judgments alone but in consultation with their physician. Again, physicians in turn rely on the considered judgments of their peers in determining standard of care under normal circumstances. In the absence of a standard of care, as with unproven treatments, the above conditions help provide a substitute, of systemic review by a group of peers and limitations on the scope of treatments to circumstances where the risk-benefit ratio would be most favourable.

### Duty to Support Learning Health Systems

Monitoring outcomes is an essential component of POEIMS. Given that, during a pandemic like COVID-19, unproven interventions are likely to be offered to multiple patients over time, medical institutions have an obligation (qua advancing the best interests of all their patients) to future patients to learn from their experiences in using the interventions. Systematic monitoring facilitates internal evaluation of safety and efficacy of a given unproven protocol, information which may inform adjustment of future treatment applications.

This is a very imperfect substitute for more rigorous evidence from clinical trials, and so institutions (and health systems more broadly) have an obligation to carry out clinical trials of unproven interventions as soon as feasible—a component of what has recently been characterized as the duty to support learning health systems (London 2021). The more solid evidence base from clinical trials can move the evidence base of a treatment from “unproven” to “proven” or “disproven,” ensuring more reliable treatments are offered to patients and removing the need for burdensome scrutiny and conditions on the provision of the treatments. Still, even if an institution is committed to supporting new clinical trials, the question remains of whether to offer unproven treatments for patients in circumstances where conducting those trials are not immediately feasible. POEIMS aims to provide oversight and rigor for care in this context.

Another component of the duty to support learning health systems is the responsibility to not impede the development or conduct of rigorous clinical trials for unproven interventions. One concern with our proposal may be that it does just that: disincentivizing clinical research. For clinicians, having an available, institutionally endorsed pathway for deployment of unproven interventions outside a clinical trial may be more appealing than the time and resources needed to set up such a trial or join one that is ongoing. Furthermore, patients who want access to innovative therapies may avoid enrolling in trials if treatments are available outside that context. This avoidance may be due to the perceived risk of being randomized to a placebo or no-treatment arm, as well as tests and procedures conducted to gather data for the purpose of research rather than clinical care.

The procedural requirements in our proposal, however, substantially limits the extent to which it would disincentivize clinical research. The burdens and inefficiencies of those procedural requirements are certainly a cost or limitation to be considered and weighed against the ethical merits, but the inefficiencies also serve as something of a silver lining. Approval through an ethics committee using POEIMS would still be on a case-by-case basis, that is, each potential recipient of an unproven intervention would need separate approval. When the potential number of patients is small, this approach may be more straightforward than an IRB-approved protocol. However, even if that process is streamlined through clearly defined eligibility criteria, it may be less efficient than a research study for larger numbers of patients, where an IRB would grant blanket approval for enrolment of all eligible patients. As a result, POEIMS may have the ancillary benefit of incentivizing utilization of clinical research at scale rather than using processes that could become cumbersome when many patients are to receive the intervention. In this way, our proposal promotes the duty to support learning health systems.

An alternative suggestion (the converse of the minimalist approach outlined above) is to much more tightly restrict the application of unproven interventions outside formal research settings (Menikoff 2021). This may better advance the duty to support learning health systems than our proposal, insofar as it would push many more treatments into research contexts that would contribute to knowledge generation and further improve healthcare and health systems. However, such restrictions may conflict with physicians’ duty of care towards individual patients. There are myriad reasons why formal research may not be established in a given context beyond clinician and patient reluctance, including the availability of resources, expertise, and institutional support. Restrictions on access do not address these barriers and so will often result in no innovative treatment being provided at all rather than an innovative treatment within an approved study protocol. Our POEIMS proposal, by contrast, provides a balance between potentially conflicting duties of care and duties to support learning health systems.

To critics of unproven interventions being provided outside the context of research whatsoever (Rid and Emanuel 2014), the overall reduction in such interventions resulting from the restrictions outlined above may well be salutary. Arguably, provision of unproven interventions does not advance physicians’ duty of care precisely because there is insufficient evidence base to confidently predict clinical benefit. However, even if that is correct, our proposal will be of use in the imperfect yet very realistic world where strict limits on provision of unproven therapies are infeasible or unlikely (whether for political or other reasons). That is to say, even if the ideal is to only provide unproven therapies in research settings, our proposal is a reasonable compromise that advances oversight of unproven therapies outside this context.

## Distinguishing POEIMS from Alternative Frameworks

Table 1 summarizes the key differences between our POEIMS proposal and that of MEURI and the FDA’s expanded access programme. While MEURI—as advanced by WHO (2016)—only requires review by an appropriately qualified ethics committee, PAHO (2020) appears to require, or at least recommends, ethics review or oversight by an REC. This requirement is similar to the FDA’s expanded access programme, which also requires IRB oversight. Our proposed framework differs from pathways that mandate IRB/REC oversight as a condition for patient use of unproven interventions during a PHEIC, as it relies on professional consensus and review by a CEC or an equivalent hospital review body.

POEIMS, therefore, would not be feasible or practical in settings where CECs have limited capacity and functions. To implement our framework, health systems would need to build up the CEC infrastructure that could provide appropriate oversight of innovative treatments, especially those which are experimental and high risk. In the Singapore context, under the recently introduced *Healthcare Services Act (2020)*, CECs in healthcare facilities will have an expanded role in reviewing the use patterns, effectiveness, and risks and benefits of selected services that are deemed higher-risk, more complex, or of greater public interest (Ministry of Health 2021). CECs that are organized and supported to carry out such functions would be appropriate bodies to review the emergency use of unproven interventions outside clinical trials during a PHEIC.

One advantage of CEC oversight is that it makes it clear that ethics review for emergency non-research use of unproven interventions differ in its aims from ethics review for use of unproven interventions in clinical trials. Under POIEMS, ethics review is a process for supporting clinicians on the difficult decision of whether to give an unproven intervention in the absence of an effective treatment. That decision is based on professional consensus on the best interest of a patient. As for the activities of monitoring, documenting, and reporting of outcomes, they are aimed at protecting the safety of the patient(s) receiving the intervention, with the *ancillary* benefit of collecting data on safety and effectiveness that could be used to inform clinical trial designs (e.g., dosage, patient population, outcome measures, etc).^2^ In other words, POIEMS makes clear that monitored emergency use of unproven interventions should not be regarded as research in its justification, purpose, and review process. Emergency use is not similar to use in formal research as its *goal* is to protect the interests and safety of patients, rather than contribute to scientific evidence or generalizable knowledge *per se*, even if it may assist with the latter by providing data to inform the design of a clinical trial.

Plausibly, the activities of monitoring, documenting, and reporting the outcomes of an unproven intervention outside of formal clinical research could be directed at both the aims of assessing patient safety and contributing to knowledge production. However, the framing of monitored emergency use as an activity aimed at knowledge generation, and of ethics review as *research ethics review*—as PAHO (2020) has recommended for the deployment of MEURI—muddies the conceptual and practical distinction between monitored emergency use and observational research. In turn, this implication raises the question of why observational research should not be conducted instead, especially when research ethics systems are not configured to review and monitor non-research uses of unproven interventions. There is a need to discuss the role and utility of observational studies on unproven interventions during a PHEIC as the data generated may be misused (Dolgin 2020; Pottegård et al. 2020; Bugin and Woodcock 2021; Annweiler, Mercat, and Souberbielle 2021). An example is a now-retracted observational study that was used to unjustifiably halt on-going clinical trials on hydroxychloroquine during COVID-19; another was used to unjustifiably endorse its widespread use (Tidey 2020). A fuller discussion on whether and how observational studies should be controlled during a PHEIC is, however, outside the scope of this paper.

Suffice to say, POEIMS would make it clear to clinicians, ethics review committees, and other relevant stakeholders why monitored emergency use is not the same as observational research. Data collected under monitored emergency use of an unproven intervention is not intended to become generalizable evidence, although they may contribute to the development of generalizable knowledge over time.

## Conclusion

In this paper, we have proposed POEIMS as an ethical alternative to the provision of experimental interventions during public health emergencies outside of formal research settings based on patient best interests and commitments to learning health systems. The framework would only be triggered in very limited circumstances where there is an urgent unmet medical need and no standard of care interventions or authorized products are available for treating the disease, and in the absence of formal clinical studies to patients to access the intervention in a supervised trial setting. In an ideal world, physicians could expeditiously initiate well-designed clinical trials to provide such interventions to their patients and collect robust data on safety and effectiveness. However, the urgency, confusion and uncertainty that typifies emergency situations, such as a global pandemic, challenges the reality. Medical doctors are not trained as researchers and their primary duties are to care for their patients. It is in this less-than-ideal messy world, where life and death decisions are routinely made, that our proposed guidance offers clinicians some professional oversight in providing interventions with uncertain safety and effectiveness according to their duty of care and patient’s best interests. Given the resources and infrastructure that POEIMS demands, its application is necessarily limited to countries or contexts that have the requisite systems in place for professional review and monitoring of outcomes. In other contexts, frameworks such as MEURI may be more appropriately applied.

## Footnotes

1 This guidance is available as part of a Working Paper Series on Pandemic Ethics published by the Centre for Biomedical Ethics, Yong Loo Lin School of Medicine, available at https://medicine.nus.edu.sg/cbme/wp-content/uploads/sites/14/2021/01/Working-paper6-2021-01-08-4.pdf. Accessed 12 March, 2021.

2 By “ancillary benefit,” we mean a benefit that is not required to justify the provision of the intervention. The unproven intervention would be provided based on the patient’s best interest whether or not the ancillary benefit was possible i.e., even if for some reason the data could never be used to inform clinical trials
